# Evaluation of the International Task Force for Disease Eradication: A Review of Past Deliberations

**DOI:** 10.4269/ajtmh.23-0885

**Published:** 2024-07-16

**Authors:** Shanze Sadiq, Ursula A. Kajani, Anyess R. Travers, Donald R. Hopkins, Frank Richards, Kashef Ijaz

**Affiliations:** The Carter Center, Atlanta, Georgia

## Abstract

The International Task Force for Disease Eradication (ITFDE) was formed at The Carter Center in 1988. Its primary purpose is to review activities and provide recommendations related to programs focused on eradication. The ITFDE also considers opportunities for disease elimination and improved control. Over the last two decades, the ITFDE has held 33 meetings, discussed 22 diseases, and made 244 recommendations. This report aims to analyze the patterns in recommendations made by the ITFDE between 2001 and 2022 and assess the ITFDE’s role, impacts, and successes in advancing elimination and eradication efforts for selected diseases. Using a thematic analysis, recommendation categories were crafted, followed by a scoping review to determine evidence of implementation for each recommendation. Categories of recommendations included research (24%), leadership (20%), medical (17%), advocacy (11%), collaboration (13%), development (8%), and financial (8%). We determined that 123 (50.4%) ITFDE recommendations were implemented in some form. Notably, the ITFDE has helped raise the profile of neglected tropical diseases. Four salient outcomes include 1) the identification of the potential eradicability of lymphatic filariasis (1993), 2) the recognition of the critical need for improved treatments of human African trypanosomiasis (2002), 3) a recommendation for the elimination of lymphatic filariasis and malaria from Hispaniola (2006), and 4) recommendations for effective and safe ways to avoid disruption of elimination and eradication programs during the COVID-19 pandemic (2020). This review of the ITFDE will help to devise new approaches to monitor its impact in the future.

## INTRODUCTION

The International Task Force for Disease Eradication (ITFDE) was formed at The Carter Center in 1988 with the Charles A. Dana Foundation’s support with the aim to evaluate disease control and prevention and the potential for eradicating selected infectious diseases. The Carter Center founder, President Carter, attended several ITFDE meetings. Both he and cofounder, Mrs. Carter, strongly supported and endorsed ITFDE recommendations. The history of the ITFDE meetings can be divided into two phases.

During the first phase of the ITFDE (1988–1992), the primary purpose was to establish the criteria to be applied systematically to determine which diseases might be eradicated. The ITFDE used the existing definition of eradication: the “reduction of the worldwide incidence of a disease to zero as a result of deliberate efforts, obviating the necessity for further control measures.” Criteria for assessing eradicability included scientific feasibility (epidemiological vulnerability, availability of effective and practical interventions, and demonstrated feasibility of elimination) and political will/popular support (perceived burden of disease, the expected cost of eradication, the synergy of eradication efforts with other interventions, and the necessity for eradication rather than control). In addition to eradicable diseases, the WHO also categorizes steps toward eradication. These labels include elimination, which is the interruption of transmission to zero incidence caused by a specific pathogen in a defined geographical area, with minimal risk of reintroduction as a result of deliberate efforts, and elimination as a public health problem, which is a term related to both infection and disease, defined by the achievement of measurable targets set by the WHO in relation to a specific disease (Table 1).

**Table 1:** Definitions and diseases discussed by the ITFDE categorized using Joint ITFDE and WHO sources

Category	Definition	Diseases*
Eradication	Permanent reduction to zero of the worldwide incidences of infection caused by a specific pathogen as a result of deliberate efforts, with no risk of reintroduction Documentation of eradication is termed *certification.*	Dracunculiasis Lymphatic Filariasis Measles Mumps Poliomyelitis Rubella *Taenia solium* Cysticercosis Yaws
Elimination (interruption of transmission)	Reduction to zero of the incidences of infection caused by a specific pathogen in a defined geographical area, with minimal risk of reintroduction, as a result of deliberate efforts: continued action to prevent reestablishment of transmission may be required Documentation of elimination of transmission is called *verification.*	Chagas Disease Human African Trypanosomiasis (*gambiense*) Leprosy Malaria Onchocerciasis Tuberculosis
Elimination as a Public Health Problem	A term related to both infection and disease, defined by the achievement of measurable targets set by the WHO in relation to a specific disease; when reached, continued action is required to maintain the targets and/or to advance interruption of transmission Documentation of elimination as a public health problem is called *validation.*	HBV/HCV Human African Trypanosomiasis (*rhodesiense*) Leishmaniasis (visceral) Schistosomiasis Soil-Transmitted Helminthiases Trachoma
Control	Reduction of disease incidence, prevalence, morbidity, and/or mortality to a locally acceptable level as a result of deliberate efforts; continued interventions are required to maintain the reduction Control may or may not be related to global targets set by the WHO.	Buruli Ulcer Leishmaniasis (cutaneous)

HBV = hepatitis B virus; HCV = hepatitis C virus; ITFDE = International Task Force for Disease Eradication.

* Categorizations not explicitly stated by ITFDE were categorized using WHO sources.

Following a review of 94 diseases, phase one of the ITFDE concluded that six diseases, dracunculiasis (Guinea worm), poliomyelitis, mumps, rubella, lymphatic filariasis, and *Taenia solium* cysticercosis, could be potentially eradicated using current technologies. It also concluded that the manifestations of seven other diseases could be eliminated: blindness from onchocerciasis, urban rabies, yaws and other endemic treponematoses, hepatitis B, neonatal tetanus, blindness from trachoma, and iodine deficiency disorders.^1^

In 2001, the Bill and Melinda Gates Foundation supported the reconvening of the ITFDE after a 9-year hiatus. The ITFDE meets twice annually at The Carter Center, which serves as the technical secretariat and primary funder for the body. Meetings are held over 1–2 days and consist of presentations by global experts followed by discussion sessions whereby recommendations are developed. The main goals of this revived task force are to review progress in the field of disease eradication, assess the status of selected diseases for control or elimination, and make recommendations regarding opportunities for eradication or better control of certain diseases. In this second and ongoing phase of the ITFDE, the task force added two diseases to its original potentially eradicable list: measles (in 2002) and yaws (in 2007).

The ITFDE has impacted the field of disease control, elimination, and eradication by issuing recommendations that have influenced organizational and individual leadership in the following ways:

- Recognizing opportunities for disease elimination, eradication, and greater control
- Identifying obstacles to elimination and eradication
- Advocating directly to policymakers of represented organizations
- Communicating lessons learned and best practices
- Providing technical assistance, encouragement, and mentorship to individuals working in the field
- Advocating to help organizations secure funding
- Publicizing successes, such as decreases in disease incidence and prevalence
- Ultimately promoting global health equity by addressing and promoting prioritization of diseases disproportionately affecting marginalized communities in low-resource settings

Notably, the ITFDE has helped raise the profile of neglected tropical diseases (NTDs) over the past two decades, with NTDs now embedded in the United Nations Sustainable Development Goals (Target 3) along with malaria, HIV/AIDs, and tuberculosis.^2^

During its second phase, 50 ITFDE members were selected from a diverse range of influential organizations, including The Carter Center, the WHO, the World Bank, UNICEF, the U.S. Centers for Disease Control and Prevention, the Pan-American Health Organization (PAHO), the Task Force for Global Health, the Bill and Melinda Gates Foundation, and several attending universities and other organizations based on specific subject area expertise. Years of involvement for members have ranged from a single year to more than 20 years. The ITFDE held 33 meetings from 2001 to 2022 to discuss the potential for and progress in controlling, eliminating, or eradicating 22 diseases and made 244 recommendations. In addition to official membership, presenters representing various nationalities, subject expertise, and national and international programs have been invited to participate in meetings according to the topic discussed.

Prior to this report, there was yet to be a review of the impact of the recommendations provided by the second phase of the ITFDE. This review aimed to analyze the patterns in recommendations made by the ITFDE over the two decades and assess the ITFDE’s role, impacts, and successes in advancing elimination and eradication efforts for selected diseases. The findings of this study were presented to the ITFDE at its 35th meeting in May 2023 and may have implications for the future strategic direction of the ITFDE.

## MATERIALS AND METHODS

A bipartite strategy was developed to evaluate the ITFDE’s work and impact. Part 1 focused on conducting a thematic analysis of the recommendations published by the ITFDE in meeting summary reports. The recommendations made by the second phase of the ITFDE were collected from the summary reports for each ITFDE meeting. Part 2 focused primarily on conducting a scoping review to measure the impact of the ITFDE by evaluating evidence of implementation of the ITFDE’s recommendations. The number of citations for each published report during this period was also calculated as a secondary measure to gauge the ITFDE’s impact.

### Part 1: Thematic analysis.

Thirty-three ITFDE meeting summary reports, published between June 2001 and March 2022, were obtained from the *Weekly Epidemiologic Record (WER)* and The Carter Center website (www.cartercenter.org).^3^^–^^35^ Recommendations, found under the “Conclusions and Recommendations” section of the reports, were identified using keywords such as “should,” “urge,” “recommend,” and “encourage.” Recommendations were extracted, organized by disease, and entered into an Excel spreadsheet for coding. All recommendations, including those discussed during the COVID-19 pandemic, were included in this review. In addition, certain diseases were only discussed as part of a group rather than individually, including measles and rubella and hepatitis C and B. They thus are addressed as a group for the purposes of discussion in this review.

Using an inductive thematic analysis, reoccurring and prevalent themes were identified and categorized into seven distinct topics: medical management, leadership, collaboration, research, advocacy, development, and financial (Table 2). Taken together, the themes encompassed the scope of the ITFDE’s work. Progress toward disease eradication, elimination, or control was assessed under these themes for each disease discussed by the task force. The distribution of recommendations across the thematic categories was examined to further understand recommendation trends within each disease.

**Table 2:** Definitions of ITFDE recommendation categories

Category	Definition
Medical	Recommendations related to treatment protocols such as prevention, diagnosis, and treatment of specific diseases
Advocacy	Related to increasing public awareness and influencing public policy, political will, funding, etc.
Research	Topics that require further research or innovation: bridging knowledge gaps about disease epidemiology, transmission, treatment, etc.
Leadership	Recommendations directed specifically to leadership entities (e.g., governments, public health agencies, nonprofit organizations, WHO, or WHO subgroups
Collaboration	Recommendations for the development of partnerships and collaborations with stakeholders, including nonprofit organizations, governments, and/or NTD programs
Development	Recommendations for the development of infrastructure and resources necessary for effective disease prevention and control, such as laboratory equipment
Financial	Identified need for financial support, need for cost-benefit assessment, or other recommendations related to increasing funding for elimination efforts

ITFDE = International Task Force for Disease Eradication; NTD = neglected tropical disease.

Descriptive statistics from the 33 ITFDE meetings were assessed using Microsoft Excel. Variables included meeting date, the diseases discussed in the respective meeting, and the total number of recommendations per meeting and disease.

### Part 2: Measuring impact.

After the thematic analysis of recommendations, a broad discovery search was conducted to identify evidence of implementation of recommendations. A search for evidence was performed separately for each unique recommendation. Search methods were tailored to the recommendation’s type, content, context, and timeline; inferred potential evidence of ITFDE’s recommendations on implementation was considered if the action occurred anytime after the meeting at which the recommendation was provided. Research-coded recommendations, for example, were evaluated by the number of articles found on PubMed, using appropriate search criteria. The number of published articles was recorded both prior to and after the year of the recommendation to determine an incremental increase after the recommendation was made; citation of ITFDE was not required for an article’s inclusion in the analysis. The WHO publication database and the *WER* were further searched for evidence of implementation when appropriate (ITFDE *WER* reports were excluded). Regarding recommendations made directly to organizations such as PAHO, authors searched the organization’s website for evidence of implementation. For nonspecific recommendations, general search engines such as Google were used to seek evidence of implementation.

To catalog evidence of implementation, codes were assigned to each recommendation indicating whether evidence of implementation was found (“yes”) or not found (“no”) or the recommendation was too broad for evidence of implementation to be distinguished (“unidentifiable”). In cases of multicomponent recommendations, if 50% or more of the components of the recommendation had evidence of implementation, they were coded as yes. The citation feature on PubMed and Scopus was further used to identify literature citing the summary reports published on each database. Furthermore, the total number of primary citations per ITFDE meeting was found via the “cited by” feature on PubMed for available summaries of the ITFDE meetings. Identical search criteria were then used in Scopus along with the cited by feature to identify additional unique citations. Owing to the constraints of the readily available and accessible information upon which this analysis was based, the sensitivity for finding evidence of implementation (i.e., yes) of ITFDE recommendations was considered low.

No statistical analysis was undertaken to explore whether the dates of disease-specific ITFDE recommendations were temporally related to trends or inflections in the topic disease’s prevalence and incidence rates; future consultation with methodologists will explore an approach to conducting such an analysis.

## RESULTS

**Figure 1.**
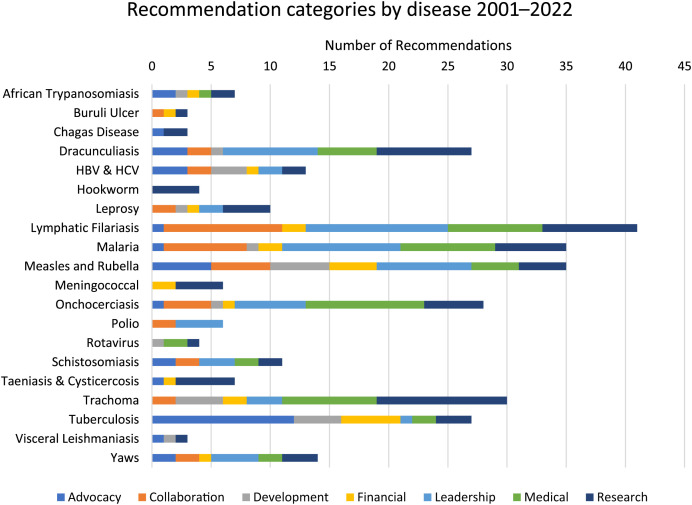
Distribution of ITFDE recommendation categories by disease. HBV = hepatitis B virus; HCV = hepatitis C virus; ITFDE = International Task Force for Disease Eradication.

The ITFDE made 244 recommendations for 22 diseases between 2001 and 2022. These recommendations were distributed among the seven themes as follows: research (24%); leadership (20%); medical (17%); advocacy (11%); collaboration (13%); development (8%); and financial (8%) (Figure 1). Research, leadership, and medical categories made up a majority of all recommendations at 61%. Research recommendations appeared for 21 of the 22 diseases included in the review. Although advocacy-related recommendations and financial recommendations had smaller shares of total recommendations, they were the second-most frequently appearing recommendation category across diseases, appearing in 15 of 22 diseases.

Lymphatic filariasis, malaria, onchocerciasis, measles and rubella, trachoma, and dracunculiasis were discussed at a greater number of meetings (e.g., “more frequently”) than other diseases and accounted for 63% of all unique recommendations made across all reports. The remaining 15 diseases were discussed at either one or two meetings between 2001 and 2022. Lymphatic filariasis was discussed the most frequently (10 occurrences) and had the greatest number of unique recommendations (33), whereas Chagas disease (2) and Buruli ulcer (2) had the least number of associated recommendations. A summary of the frequency of discussion and the total number of recommendations across all reports for all diseases is provided in Table 3.

**Table 3:** Frequency of topic recurrence and number of ITFDE recommendations by disease 2001–2022

Disease	Frequency of Topic Recurrence	Total Recommendations	Dates Mentioned
Lymphatic Filariasis*	10	32	10/18/2002, 10/11/2007, 10/29/2008, 10/12/2010, 4/6/2011, 11/27/2012, 1/14/2014, 11/8/2016, 10/20/2020, 5/4/2021
Malaria	8	24	10/4/2005, 5/12/2006, 10/11/2007, 10/29/2008, 10/12/2010, 11/27/2012, 10/20/2020, 5/4/2021
Measles/Rubella*	5	30	1/25/2002, 6/4/2009, 11/10/2015, 10/22/2019, 10/20/2020
Dracunculiasis*	5	22	10/16/2003, 10/29/2008, 4/28/2015, 10/17/2017, 10/20/2020
Onchocerciasis	5	20	6/18/2001, 1/11/2007, 4/6/2011, 1/14/2014, 10/20/2020
Trachoma	4	27	1/19/2005, 10/12/2010, 11/8/2016, 10/20/2020
Tuberculosis	2	24	1/12/2010, 3/14/2022
Yaws*	2	8	11/10/2007, 11/27/2012
Schistosomiasis	2	7	6/18/2001, 4/12/2012
Leprosy	2	6	18/06/2001, 4/23/2018
Taeniasis/Cysticercosis*	2	6	4/16/2003, 10/07/2013
Meningococcal Meningitis	2	4	4/16/2003, 7/10/2013
Buruli Ulcer	2	2	10/11/2007, 10/29/2008
HBV/HCV	1	9	6/20/2017
African Trypanosomiasis	1	5	10/18/2002
Hookworm	1	4	3/24/2004
Rotavirus	1	4	10/30/2009
Polio*	1	4	10/20/2020
Visceral Leishmaniasis	1	3	3/24/2004
Chagas Disease	1	2	6/18/2001

HBV = hepatitis B virus; HCV = hepatitis C virus; ITFDE = International Task Force for Disease Eradication.

* These diseases were declared eradicable by the ITFDE. Polio and mumps were identified as eradicable in the first phase of the ITFDE.

During the impact evaluation, 123 (50.4%) of the 244 recommendations were categorized as having evidence of implementation, whereas 75 (30.7%) were categorized as no evidence of implementation detected and 46 (18.9%) were categorized as unidentifiable because of the lack of specificity within the language of the recommendation. Seventy-nine percent of research-related recommendations had evidence of implementation, the largest among all recommendation categories. Advocacy-related recommendations had the least amount of identifiable evidence at only 20%. The remaining categories were split evenly between evidence of implementation, no evidence, and unidentifiable. Table 4 gives a comparative breakdown of the evidence of implementation sorted by thematic categories.

**Table 4:** Evidence of implementation by thematic category

Category	Total No. of Recommendations per Category*	Evidence of Implementation		
		Yes	No	Unidentifiable
Advocacy	35 (11%)	7	14	14
Collaboration	41 (13%)	21	12	8
Development	23 (8%)	16	7	0
Financial	24 (8%)	12	10	2
Leadership	63 (20%)	24	27	12
Medical	52 (17%)	25	12	15
Research	76 (24%)	60	13	3
Total	314	165	95	54
Total as %	–	52.5	30.3	17.2

* Recommendations were categorized under multiple relevant categories, increasing the total number of recommendations from 244 to 314 per category.

**Figure 2.**
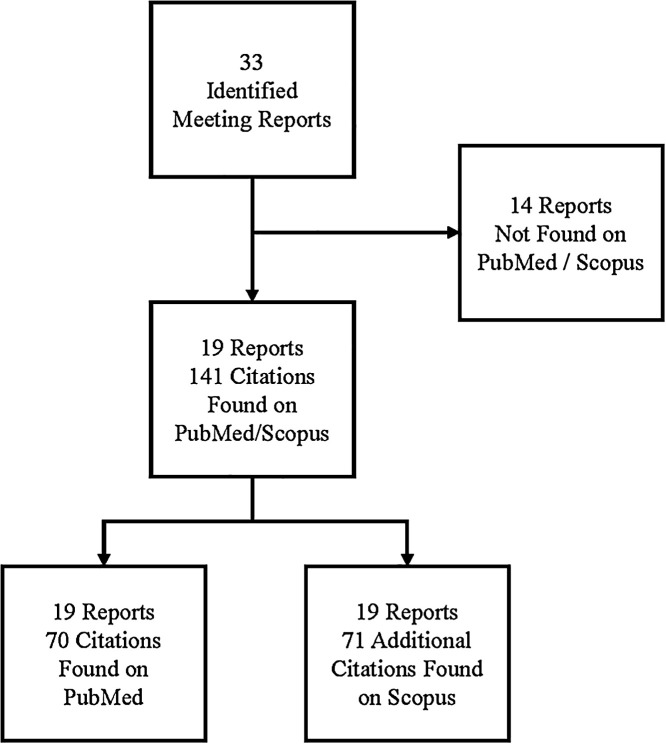
Flow chart of identified primary citations.

Lastly, 19 of the 33 ITFDE meeting summaries were available on PubMed or Scopus, from which 141 citations were found. Of the 141 citations, seven citations were duplicate results in which a source cited more than one meeting and 134 were unique citations across the 19 meeting summaries (Figure 2). Citations per meeting ranged from 0 citations for the October 2009 meeting to 26 citations for the January 2007 meeting.

## DISCUSSION

In the 33 meeting summary reports published by the ITFDE between June 2001 and March 2022, the task force discussed 22 diseases and made 244 recommendations. Although lymphatic filariasis was discussed the most often and had the greatest number of recommendations, most of these discussions and recommendations overlapped with malaria and onchocerciasis, both of which share intervention tools in Africa with lymphatic filariasis (long-lasting insecticidal bed nets and ivermectin mass drug administration [MDA]). Fewer than 10 recommendations addressed lymphatic filariasis alone. Moreover, only seven diseases (lymphatic filariasis, malaria, onchocerciasis, measles, rubella, trachoma, and dracunculiasis) were discussed more than twice. Of these diseases, lymphatic filariasis, measles, rubella, and dracunculiasis were identified by the ITFDE as eradicable, whereas malaria, onchocerciasis, and trachoma were identified by the ITFDE as eliminable. Although there may be a pattern in the ITFDE’s choice to focus more on diseases that it felt could be eradicated or eliminated, there were important exceptions to this trend, such as polio, yaws, taeniasis/cysticercosis, hepatitis B and C, and Chagas disease, which were discussed infrequently. Moreover, although the ITFDE initially identified mumps as one of eight eradicable diseases, it has never been the focus of a separate ITFDE meeting, and the task force has not made any recommendations regarding efforts toward eradicating mumps. Similarly, polio was also identified as an eradicable disease but was only discussed once during the second phase of ITFDE in the context of restrictions caused by the global COVID-19 pandemic. The ITFDE decided at the beginning of its second phase that it would not routinely review polio, as this well-funded eradication program was supported by extensive and continual oversight by other bodies.

The analysis found research to be the most frequently recommended category by ITFDE, accounting for approximately half of the 124 recommendations for which evidence of implementation was found. Research recommendations generally focused on defining areas urgently requiring more understanding. This trend is likely due to the fact that most of the 23 diseases being discussed were still in the process of developing effective disease control and elimination strategies, a stage when additional research was required. In contrast, recommendations in the later stages focused on implementation challenges, such as the logistics of interventions and the need for strengthened leadership. For example, recommendations made by the ITFDE in 2001 for onchocerciasis included prioritizing research on an effective macrofilaricide and the significance of low-transmission levels, whereas in more recent years, such as 2014, the ITFDE suggested that African countries would benefit from “authoritative, clear, sound, and timely advice” on effective technical strategies for elimination of onchocerciasis from the continent.^16^ Furthermore, advocacy-related recommendations appeared in almost all disease recommendations. Nearly half of all recommendations in each category had no or unidentifiable evidence of implementation. Therefore, it would be prudent for the ITFDE to understand and maximize factors that affect the implementation of recommendations, such as how information is disseminated to the global public health community and whom the ITFDE is addressing in each recommendation.

Tuberculosis had an uncharacteristically large number of recommendations compared with other diseases of its category (i.e., diseases the ITFDE identified as not eradicable or not eradicable now). Tuberculosis had 24 recommendations across two discussions, whereas leprosy, for example, was discussed twice and had only five recommendations. This may be because the ITFDE meeting on TB in March 2022 fell a week before World TB Day, which may have also influenced the focus on advocacy within the recommendations. In fact, 33% of all ITFDE TB recommendations focused on advocacy, whereas in other ITFDE meetings, advocacy represented a much smaller proportion of overall recommendations, accounting for only 12% of all recommendations.

In addition to the evidence for implementing various recommendations, the ITFDE has had several noteworthy impacts in its goal to reduce human suffering, whether by disease eradication, elimination, or control. The ITFDE was the first international body to assert that it was possible to eradicate lymphatic filariasis using currently available tools in 1993.^1^ That helped to engender an international MDA and palliative care campaign led by the WHO that delivered more than 9 billion cumulative treatments by MDA to more than 935 million persons in 2000–2021. In 2021, 17 of the 72 lymphatic filariasis–endemic countries had been validated by the WHO as having eliminated lymphatic filariasis as a public health problem.^36^ Furthermore, in 2002, the ITFDE noted a critical need for improved treatments of human African trypanosomiasis (HAT), which has seen significant progress over the past two decades and has culminated in the registration of the first oral drug, fexinidazole, for treating this disease.^30^ The incidence of Gambian-type HAT, which was 97% of all cases and affected an estimated 300,000–600,000 persons in 2002, has been reduced dramatically to fewer than 1,000 cases reported annually.^37^

In 2006, the ITFDE concluded that the elimination of malaria and lymphatic filariasis from the island of Hispaniola was “technically feasible, medically desirable, and would be economically beneficial to both the Dominican Republic and Haiti.”^2^ This spurred the Hispaniola Initiative to be launched by The Carter Center, which has which has assisted the ministries of health in reducing malaria cases on the island by 87% since 2008 and reaching criteria for stopping lymphatic filariasis MDA in 86% of the 140 lymphatic filariasis–endemic districts in Haiti and 100% of the affected districts in the Dominican Republic by 2021.^38^ Recently, in 2020, the ITFDE discussed the impact of the COVID-19 pandemic on eradication/elimination programs, including reviewing program challenges and providing specific recommendations for dracunculiasis and polio eradication, as well as for measles, rubella, malaria, onchocerciasis, lymphatic filariasis, and trachoma elimination.^24^ These recommendations provided guidance during great uncertainty and risk for MDA and mass vaccination programs.

The observed trends in the distribution of recommendation categories speak to what the ITFDE has historically focused on and prioritized. When future strategic direction of the ITFDE is devised, these observations may be helpful in considering new topics that could impact the successful implementation of eradication, elimination, and control efforts such as mental health in populations affected by NTDs and climate change. Other future directions might include revising the ITFDE membership criteria and seeking better ways to increase and monitor the impact of ITFDE’s recommendations.

### Limitations.

This report provides basic descriptive data about ITFDE deliberations without any attempt at statistical analyses. Of 33 papers, 19 were accessible through PubMed or Scopus, with reports missing from January 2001 to October 2005 and from April 2018 to March 2022. The lack of availability of these reports may indicate limitations in disseminating recommendations. This review refrains from attempting to measure direct correlations between ITFDE’s recommendations and progress in disease control and elimination efforts. The review used an insensitive approach to detect evidence of implementation for each recommendation using a limited search on readily available online sources. Once evidence was found for a particular recommendation, additional research was not continued. Furthermore, evidence for certain categories, such as research-related recommendations, may have been more easily detectable through a scoping review than others, such as advocacy, leadership, or logistics. The inclusion of formal qualitative methods, including interviews with country and global experts and health professionals, may be used in the future to broaden the evidence identification process. In addition, the wording and structuring of recommendations were not assessed, including the recommendations’ scope or whether they addressed a particular organization, which may have also influenced the degree of evidence of implementation found. However, these initial findings lay the groundwork for developing new retrospective and prospective approaches through which the aforementioned limitations may be addressed and expanded upon.

## CONCLUSION

The impact of the ITFDE on global disease eradication, elimination, and control efforts had not been assessed before the undertaking of this review. Although further research is needed to draw more definitive conclusions, the preliminary findings and anecdotal evidence found here suggest that the ITFDE has made several noteworthy impacts on the progression of global disease eradication and elimination efforts. These findings will be valuable in framing the ITFDE’s strategic direction, including which diseases to discuss in the future. Study results may also help to devise strategies to increase the ITFDE’s impact and extend its influence on the global public health and disease control community.
